# Blood–brain barrier opening in a large animal model using closed-loop microbubble cavitation-based feedback control of focused ultrasound sonication

**DOI:** 10.1038/s41598-022-20568-y

**Published:** 2022-09-27

**Authors:** Chih-Yen Chien, Lu Xu, Christopher Pham Pacia, Yimei Yue, Hong Chen

**Affiliations:** 1 Department of Biomedical Engineering, Washington University in St. Louis, Saint Louis, MO 63130 USA; 2 Department of Radiation Oncology, Washington University School of Medicine, 4511 Forest Park Ave., Saint Louis, MO 63108 USA

**Keywords:** Biological techniques, Biotechnology

## Abstract

Focused ultrasound (FUS) in combination with microbubbles has been established as a promising technique for noninvasive and localized Blood–brain barrier (BBB) opening. Real-time passive cavitation detection (PCD)-based feedback control of the FUS sonication is critical to ensure effective BBB opening without causing hemorrhage. This study evaluated the performance of a closed-loop feedback controller in a porcine model. Calibration of the baseline cavitation level was performed for each targeted brain location by a FUS sonication in the presence of intravenously injected microbubbles at a low acoustic pressure without inducing BBB opening. The target cavitation level (TCL) was defined for each target based on the baseline cavitation level. FUS treatment was then performed under real-time PCD-based feedback controller to maintain the cavitation level at the TCL. After FUS treatment, contrast-enhanced MRI and ex vivo histological staining were performed to evaluate the BBB permeability and safety. Safe and effective BBB opening was achieved with the BBB opening volume increased from 3.8 ± 0.7 to 53.6 ± 23.3 mm^3^ as the TCL was increased from 0.25 to 1 dB. This study validated that effective and safe FUS-induced BBB opening in a large animal model can be achieved with closed-loop feedback control of the FUS sonication.

## Introduction

FUS-induced blood–brain barrier opening (FUS-BBBO) is a promising technique for brain drug delivery by disrupting the BBB to enable the delivery of therapeutic agents from the blood circulation to the brain. Successful brain drug delivery has been demonstrated in small animals^1–6^, large animals^7–10^, and patients^11–18^. More than ten clinical trials are currently actively recruiting to evaluate the application of FUS-BBBO in patients with various brain diseases, such as glioblastoma and Alzheimer's disease (https://clinicaltrials.gov/). One critical challenge in the clinical application of FUS-BBBO is the inter- and intra-variability of the treatment. Variations in the acoustic pressure and microbubble size distribution and concentration within the FUS focal region among patients or among different brain locations for a single patient contribute to the inter- and intra-variability of the treatment^19–21^. The in situ acoustic pressure varies due to skull heterogeneity and variation in the incident angle of the FUS beam relative to the skull^22,23^. The in situ microbubble concentration distribution in the targeted brain region varies due to differences in factors, such as vascular density, vessel size, and blood flow^19,20^.

Passive cavitation detection (PCD)-based feedback control has been developed for real-time detection of microbubble cavitation and feedback control of the FUS sonication pressure^12,24–30^. Several previous publications proposed to control cavitation activity by using feedback control algorithms that considered the individual differences in the detected cavitation signals. The approach proposed by O'Reilly et al^28^ and later adopted in the clinical trials defined the targeted cavitation level (TCL) based on calibration performed with FUS sonication in the presence of microbubbles for an individual subject. The calibration required ramping up the pressure to an upper threshold when ultra-harmonic emission was detected and then maintaining the pressure at a fixed percentage of the upper threshold (e.g., 50%). Although this approach has already been used in clinical studies, the need to reach the upper threshold for the calibration increases the risk of generating inertial cavitation. Inertial cavitation is known to be associated with the risk of causing brain tissue damage^31^. Others have proposed an alternative strategy to avoid overexposure by defining the TCL relative to the baseline stable cavitation level detected before microbubble injection^26,27^. This strategy did not take into consideration of variations in microbubbles within the focal region (e.g., variations in microbubble concentration and size distribution).

In our previous study^32^, we proposed a closed-loop feedback control algorithm for FUS-BBBO with the TCL defined based on the baseline stable cavitation level of each subject acquired by "dummy" FUS sonication. The dummy sonication applied a low acoustic pressure for a short duration at the targeted brain location in the presence of microbubbles to acquire the baseline cavitation signal. The baseline cavitation level considered the cavitation signal differences in individual target, which were affected by the in situ acoustic pressure and microbubble distribution within the targeted location. The TCL was defined relative to the baseline stable cavitation level (e.g., 0.5 dB above the baseline). FUS treatment was then performed under real-time control of the acoustic pressure to maintain the stable cavitation level at the selected TCL. The performance of the proposed feedback control algorithm was evaluated in a mouse model. The results showed that the proposed algorithm had high stability and successfully controlled the FUS-BBBO delivery outcomes at selected TCL^32^.

Studies using a large animal model are needed to evaluate the clinical translatability of PCD-based feedback control algorithms. Although multiple feedback control algorithms have been proposed, most approaches, including ours^32^, were only assessed in small animal models. Only a few studies were performed using large animal models. Kamimura et al. evaluated their feedback controller in non-human primates^26^. Their feedback controller defined the TCL as the ratio of the signal power spectrum after microbubble injection and the corresponding baseline power spectrum before microbubble injection for each subject. Later, the same group optimized their feedback controller by using an intra-pulse analysis to achieve precise control of the stable cavitation during the sonication of each FUS pulse. They showed that their optimized controller increased the sensitivity in detecting sudden changes in microbubble cavitation in non-human primates^33^. More studies in large animal models are needed to accelerate the clinical translation of PCD-based feedback control algorithms.

The objective of this study was to evaluate the feasibility of our PCD-based feedback controller in controlling FUS-BBBO in a porcine model. The porcine model was selected because of its similarity in blood volume/body weight, skull thickness, and brain morphology to humans^34,35^. It also has less ethical concerns and is easier to access compared with the non-human primate model. A total of 3 pigs, with each pig sonicated at multiple brain locations (3–5 targets), were used to evaluate the FUS-BBBO outcome under three different TCLs. FUS-BBBO outcome was assessed by contrast-enhanced MRI based on the extravasation of the MRI contrast agent. The safety of FUS-BBBO at each TCL was evaluated by in vivo MRI scans and ex vivo histological analysis.

## Material and methods

### Animal preparation

All animal procedures were reviewed and approved by the Institutional Animal Care and Use Committee at Washington University in St. Louis in accordance with the Guide for the Care and Use of Laboratory Animals and the Animal Welfare Act. A total of 3 pigs (age: around 4 weeks old; sex: male) with each pig sonicated at multiple brain locations (3–5 targets) in the cortical brain region were used in this study to evaluate three different TCLs using the proposed PCD-based feedback control algorithm. The standard operation procedure (SOP) for performing FUS-BBBO in pigs was established in our previous work^36^. Following our reported SOP, pigs were sedated with an intramuscular injection of ketamine (2 mg/kg), xylazine (2 mg/kg) and telazol (4 mg/kg), intubated, and maintained under general anesthesia using isoflurane and positive pressure ventilation. The hair on the pig head was removed using depilatory cream (Nair, Church & Dwight Co., Princeton, NJ). The shaved section was covered with a non-toxic, water-soluble ultrasound gel to ensure optimal acoustic coupling. A catheter was placed in the ear vein for microbubble and MRI contrast agent injections. A fiber-optic pulse oximeter (Nonin 7500FO, Plymouth, MN) was used to monitor the blood oxygen level and pulse rate during the procedure. The animal's body temperature was monitored and maintained with heated blankets. This study was carried out in compliance with the Animal Research: Reporting of In Vivo Experiments guidelines.

### Experiment setup

The ultrasound system was adopted from our previous study^32^ and modified accordingly. A single-element FUS transducer with an aperture of 75 mm, a radius of curvature of 60 mm, and a center opening of 25 mm in diameter was used in this study. The FUS transducer was coupled to a 3D-printed cone filled with degassed and distilled water and attached to a 3D stage motor (IGT, Bordeaux, France) for mechanical movements. The motor was controlled by the custom MATLAB script to move the FUS transducer to different brain locations. The FUS transducer was impedance matched to be operated at 500 kHz. It was driven by an arbitrary waveform generator (Agilent 33500B; Agilent Technologies, Loveland, CO, USA) that was connected to a 53-dB power amplifier (1020 L; E&I, Rochester, NY, USA). The acoustic pressure fields generated by the FUS transducer were calibrated using a needle hydrophone (HNP-0200; Onda Inc., Sunnyvale, USA) in a degassed water tank. The axial and lateral full-width-at-half-maximum (FWHM) dimensions of the FUS transducer were 26.7 mm and 3.7 mm, respectively. The peak negative pressures of the FUS transducer at different voltage input levels were measured at the focus of the transducer in a water tank. A single-element ultrasound transducer (V323, Olympus, Tokyo, Japan) with a center frequency of 2.25 MHz and a bandwidth (− 6 dB) of 600 kHz was used as inserted through the center hole of the FUS transducer and confocally aligned with the FUS transducer using a 3D-printed housing. This transducer was used as a passive cavitation detector to acquire cavitation emissions from the microbubbles during FUS sonication. It was connected to a PicoScope (5244B, Pico Technology, Cambridgeshire, UK) for PCD data acquisition. The data acquisition was synchronized with the FUS sonication by using the trigger-out signal of the arbitrary waveform generator to trigger the PicoScope. The signal acquired by the PCD was sampled at 40 MHz. All the equipment was controlled by a personal computer using a custom MATLAB program.

**Figure 1:**
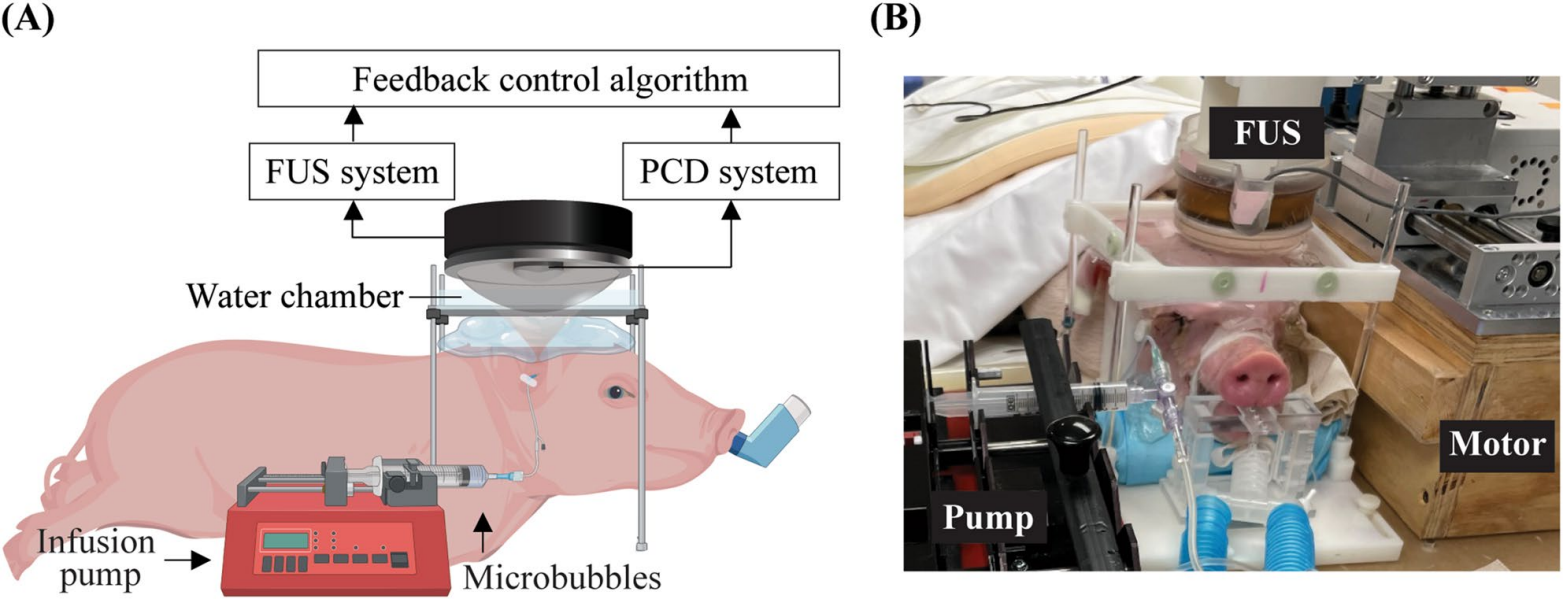
Experiment setup for in vivo experiment (**A**) Illustration of the feedback-controlled FUS system. The experiment setup was composed of three parts: (1) FUS system: FUS transducer, function generator, and power amplifier. (2) PCD system: PCD, pre-amplifier, and PicoScope. (3) Feedback control algorithm: a customized MATLAB program for the feedback control. (**B**) Picture of the experiment setup during the pig study. The FUS transducer was connected to a 3D stage for positioning.

The experiment setup for the pig study is shown in Fig. 1. The transducer was coupled to the pig head through a water chamber. The pig's head was supported and stabilized by a bite bar and two supports on the left and right sides. A surface leveler was used to guide the positioning of the pig head such that the top of the skull was level with the operating table. A neuronavigation system (BrainSight; Rogue Research Inc., Montreal, QC, Canada) was used to guide the positioning of the FUS transducer for targeting a specific brain location. Definity microbubbles at a dose of 10 μl/kg were injected intravenously through the ear vein catheter. The injection was performed using a syringe pump. Microbubble infusion was started 20 s before FUS sonication began to allow microbubbles to flow through the ear vein catheter and reach the pig brain. The infusion lasted until the end of sonication at a constant rate of 1.67 mL/min. All pigs were treated by FUS with output pressure controlled in real-time using the proposed PCD-based closed-loop feedback control algorithm.

### FUS-BBBO under real-time closed-loop feedback control

Details of the proposed real-time PCD-based feedback-controlled FUS-BBBO procedure were described in our previous publication^32^. Briefly, the proposed feedback control algorithm was composed of two steps:

*Step 1:* Dummy FUS sonication was used to establish the baseline stable cavitation (SC) level for each individual sonication after microbubble infusion started. FUS sonication was performed with a center frequency of 500 kHz, a pulse repetition frequency of 1 Hz, a pulse length of 10 ms (i.e., duty cycle: 1%) for 5 s. The dummy FUS sonication was performed at 0.3 MPa (free-field acoustic pressure). Under such low FUS acoustic pressure and short sonication duration, no BBB opening was induced, while individual differences in the detected baseline cavitation signal were captured. During the sonication by each FUS pulse, acoustic emission from microbubbles was recorded by the PCD and processed by the Fast-Fourier transform (FFT) algorithm. The SC level was calculated by summing the magnitude of the spectrum within a ± 0.02 MHz bandwidth centered at the fourth harmonic (i.e., 2 MHz) of the FUS transducer. The fourth harmonic emission was chosen because it represents the SC activities of microbubbles, and 2 MHz was the closest harmonic frequency to the center frequency of the PCD transducer. Five PCD signals were acquired, and the average SC level calculated from these signals was used to define the baseline SC level.

*Step 2:* Real-time feedback-controlled FUS-BBBO. During FUS sonication with microbubble infusion, cavitation was monitored by PCD in real-time, and a custom closed-loop feedback control algorithm was used to tune FUS acoustic pressure to maintain the SC level at different TCLs that were defined to be 0.25 dB, 0.5 dB, or 1 dB above the baseline SC level for 180 s. Different TCLs were used to investigate the dependencies of FUS-BBBO drug delivery outcome and safety on the TCL. The proposed feedback control algorithm consisted of two sonication phases: the ramping-up phase and maintaining phase. The ramping-up phase increased FUS acoustic pressure pulse by pulse until the SC level reached the TCL. Then the control algorithm switched to the maintaining phase with the acoustic pressure adjusted to maintain the SC level within the target range (i.e., TCL ± tolerance range) until the end of the sonication. If the SC level was located within the range of TCL ± tolerance range, the FUS output pressure was kept the same. FUS output pressure of the next pulse was decreased or increased immediately for the case that the SC level was higher or lower than TCL ± tolerance range.

### Feedback controller characterization

The stability of the feedback control algorithm was determined by the good pulse rate, which measured the percentage of FUS pulses during which the SC level was within the target range in the maintaining phase. In other words, good pulse rate counts the numbers of pulses within target range over all pulses in the maintaining phase. A higher good pulse rate represents higher controllability of the cavitation activities.)

### Inertial cavitation monitoring

Inertial cavitation (IC) level was quantified based on the acquired cavitation signals to serve as a safety check during sonication. IC level was calculated by summing the magnitude of the spectrum within 2.1 ± 0.02 MHz. These frequencies were chosen to quantify the level of the broadband signals by avoiding harmonics and ultra-harmonics. The presence of an IC event was defined when the IC level was over 1 dB above the baseline IC level quantified based on the signals acquired during dummy FUS sonication. IC probability was calculated by the percentage of IC events that were present during the maintaining phase. Higher IC probability indicates a higher occurrence rate of IC events and suggests a higher potential for tissue damage^31,37–39^.

### Controlled FUS-BBBO outcome assessment

T1-weighted Gradient Echo MRI scans (Scan resolution: 0.85 × 0.85 mm, slice thickness: 1 mm; TR = 8.16 ms, TE = 3.76 ms, Acqsuition matrix: 152/150, flip angle: 10 deg) were acquired both before and after FUS treatment to assess the BBB permeability at each TCL. Each TCL was evaluated at 4 targeted brain locations. The T1-weighted images of pre- and post-FUS at each target were compared using a custom MATLAB script as previously described^36^ to define BBB opening. Briefly, the analysis was started by defining an elliptical region of interest (ROI, major axis: 19 mm; minor axis: 8 mm) in the FUS-treated site and non-treated site. The ventricles were avoided in both ROIs because the hyperintensity of the MR contrast agent in the ventricles would confound the calculation of hyper-enhancement in the tissue due to BBB opening. Next, a voxel in the ROI was considered to represent BBB opening if the voxel intensity within the FUS-treated ROI was greater than 3 × standard deviations above the mean intensity of the non-treated ROI. Dimension for the individual voxel is 0.85 mm × 0.85 mm × 1 mm. The contrast enhancement volume was then estimated by calculating the sum of all identified voxels for each image slice. The slice with the max contrast enhancement was selected to represent the volume of BBB opening for each target.

### Safety analysis

In vivo* safety assessment.* The safety of different TCLs was evaluated with a T2*-weighted MRI scan (with the same parameters as the pre-treatment T2*-weighted sequence) to detect FUS-induced hemorrhages after the FUS-BBBO procedure. Hemorrhages would appear as hypo-intensity spots on the T2*-weighed images.

Ex vivo* safety assessment.* The pigs were euthanized, and their brains were harvested after the MRI scans were completed. Each brain was fixed for 1 week in 10% formalin. The whole brain was placed in a 3D-printed brain slicing matrix and sectioned horizontally into 4-mm thick slabs around the FUS treatment area. The 4-mm thick slabs were immersed in 15% sucrose overnight followed by 30% sucrose, and then cryosectioned into 10 µm slices for hematoxylin and eosin (H&E) staining to examine the area covered by red blood cells. Digital images of tissue sections were obtained using an all-in-one microscope (BZ-X810, Keyence, Osaka, Japan). The total area covered by red blood cells was calculated by summing all the pixels with the red hue in the ROI that covered the FUS-treated site. The non-treated site was used as the control.

### Statistical analysis

Statistical analyses were performed using GraphPad Prism (Version 9.0, La Jolla, CA, USA). Differences among multiple groups were determined using one-way ANOVA followed by the Tukey's test for group-wise comparisons. We performed a normality test before ANOVA. A one-sample t-test was performed if the samples did not pass the normality test. *P* value < 0.05 was used to determine statistical significance. *P* value < 0.05 was used to determine statistical significance.

## Results

### Performance of the proposed feedback control algorithm in pigs

**Figure 2:**
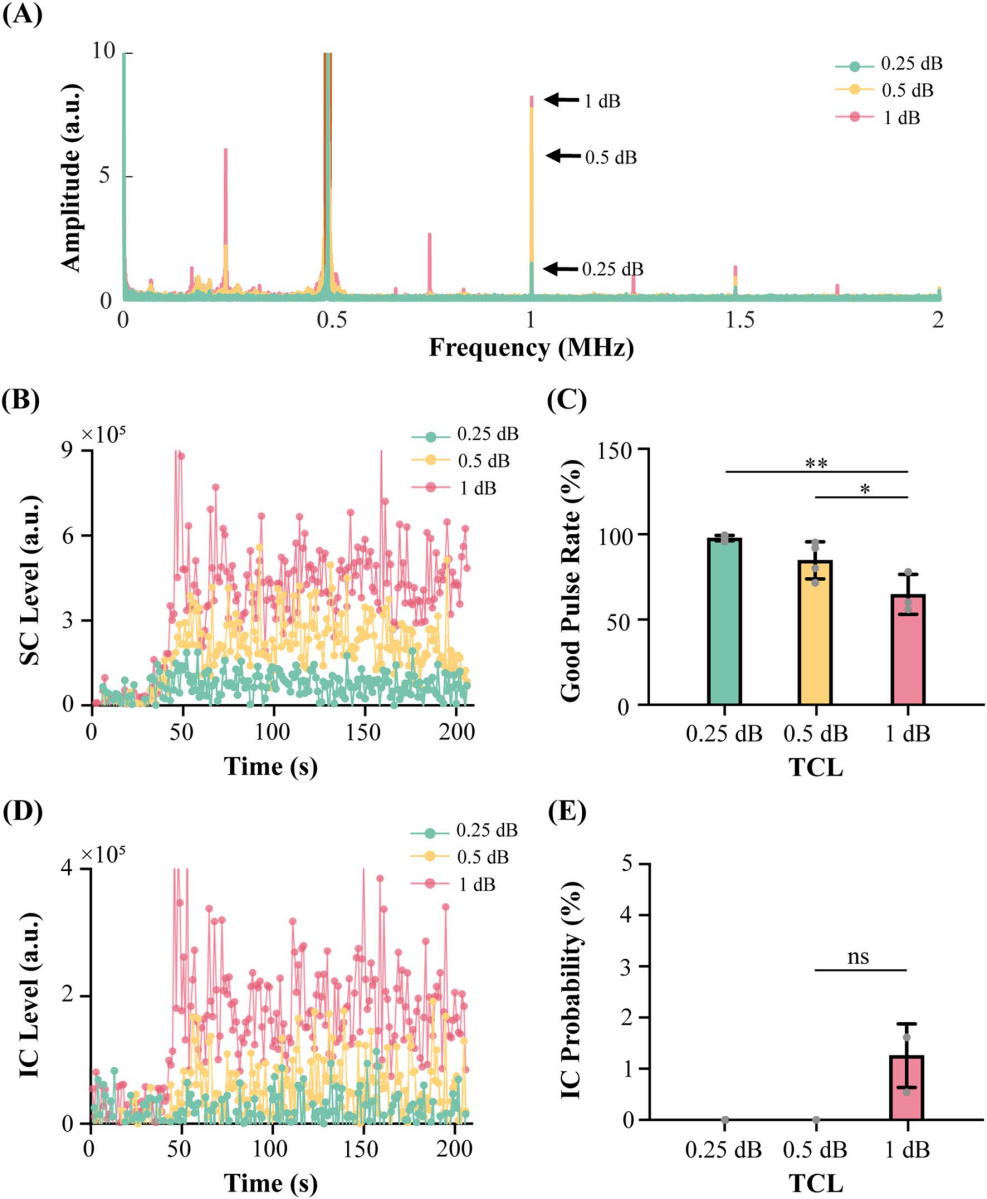
Performance of the proposed feedback control algorithm for in vivo experiment. (**A**) Representative spectra at different TCLs (i.e., 0.25 dB, 0.5 dB, or 1 dB above the baseline SC level). (**B**) SC level as a function of time at different TCLs. Each color represents the average SC level for each TCL group. (**C**) Good pulse rate of the feedback control algorithm at each TCL. (**D**) The average IC level at each TCL. (**E**) IC probability at different TCLs. The bar plot in (**C**) and (**E**) shows the mean and standard deviation. Each circular point represents the result obtained from each target. (Tukey’s test *P < 0.05; **P < 0.01; ****P < 0.0001; ns/no label: not significant).

The proposed feedback control algorithm was successfully applied to the pig model. Figure 2A shows the representative spectrum at each TCL. Higher amplitude of spectrum was observed at higher TCL. Ultra-harmonic emission was observed at TCL of 1 dB, and sub-harmonic emission was observed at TCL >  = 0.5 dB. The plot of the mean SC levels during sonications (Fig. 2B) shows that the proposed feedback control algorithm was capable of controlling the FUS sonication to maintain the SC level at each TCL. For the TCL of 1 dB group, one PCD dataset was not included because the PCD data was not saved due to operational error. The stability of the control algorithm, as measured by the good pulse rate (Fig. 2C), was 97.7%, 84.7%, and 64.7% on average for TCL = 0.25 dB, 0.5 dB, and 1 dB, respectively.

### Inertial cavitation monitoring

The measured mean IC levels for each group are presented in Fig. 2D. Average IC probability was 0%, 0%, and 1.25% for TCL of 0.25 dB, 0.5 dB, and 1 dB, respectively (Fig. 2E). No significant difference was observed in the IC probability among different TCL groups.

### Controlled FUS-BBBO outcome

**Figure 3:**
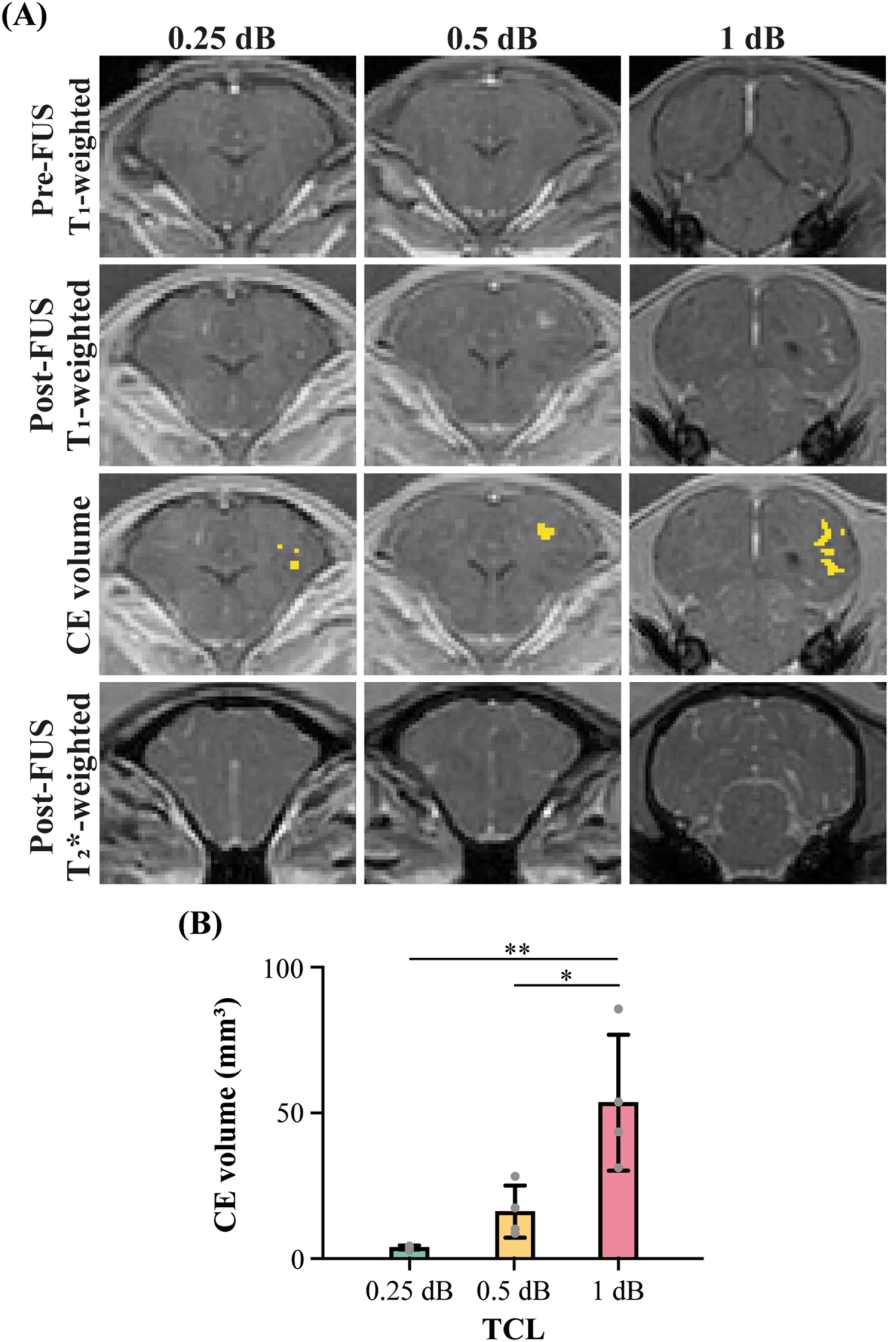
The proposed feedback controller achieved different levels of BBB opening in pigs. (**A**) Representative contrast-enhanced (CE) T1-weighted MRI scans were acquired before (first column) and after FUS (second column) to quantify CE volume (i.e., yellow-shaded area in the third column). The representative T2*-weighted MRIs acquired at the same position as the T1-weighted MRIs (bottom column) (**B**) The CE volume at each TCL. The bar plot in (**B**) shows the mean and standard deviation. Each circular point represents the result obtained from each target. (Tukey’s test *P < 0.05; **P < 0.01; ***P < 0.001).

Figure 3A presents representative T_1_-weighted MRI images that show the extravasation of the MRI contrast agent before (first column) and after (second column) applying FUS sonication at TCL of 0.25 dB, 0.5 dB, and 1 dB, respectively. Yellow-color highlighted area in the third column of Fig. 3A indicates the BBB opening area. The quantified BBB opening volume in the FUS-treated area are 3.8 ± 0.7 mm^3^, 16.2 ± 9.0 mm^3^, and 53.6 ± 23.3 mm^3^ on average for TCL of 0.25 dB, 0.5 dB, and 1 dB, respectively (Fig. 3B). These data showed that the average contrast enhancement volume monotonically increased as the TCL increased from 0.25 dB to 1 dB.

### Safety evaluation

**Figure 4:**
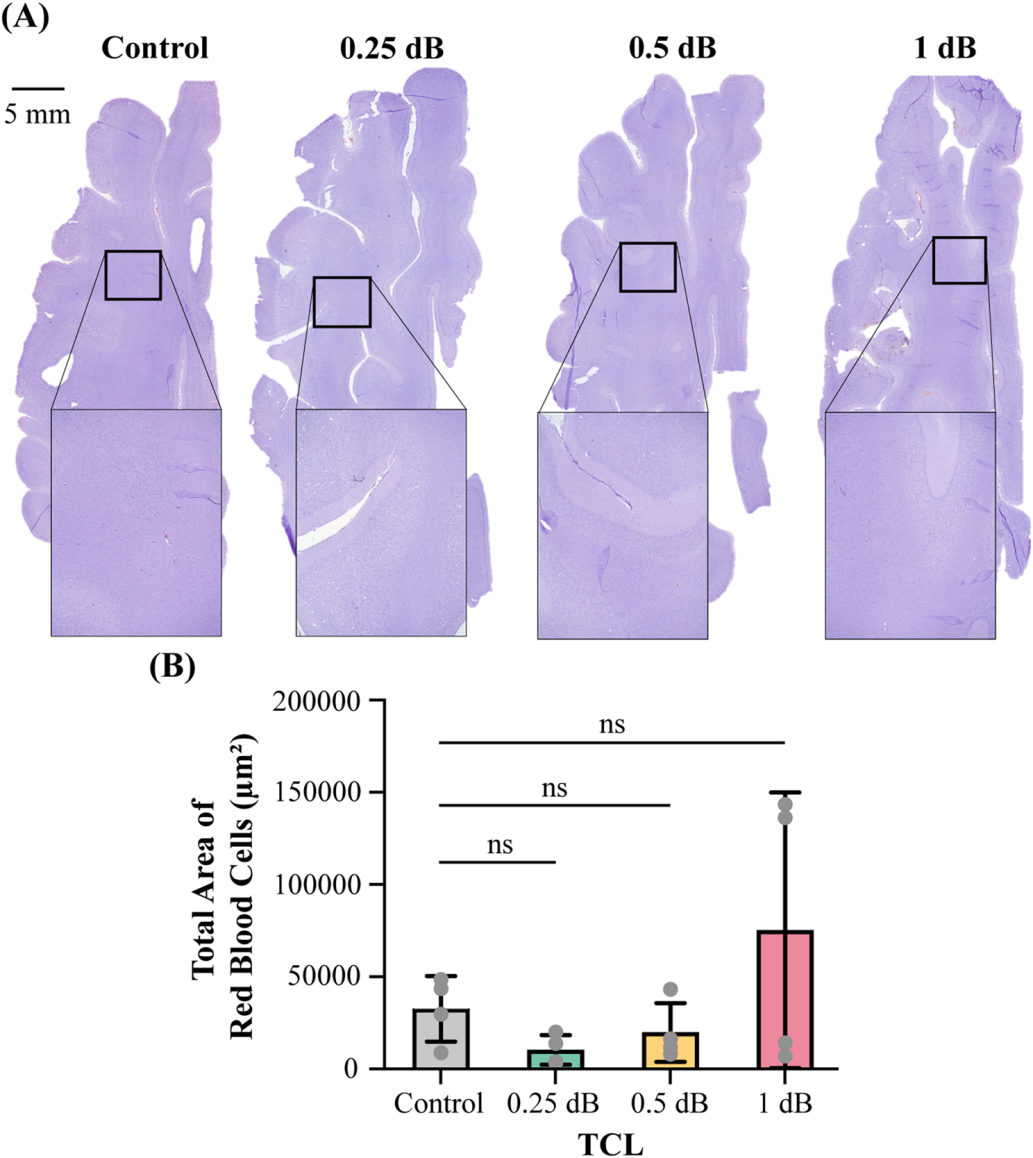
Safety evaluation of feedback-controlled FUS-BBBO in pig studies. (**A**) Representative H&E staining of the FUS-treated  site at different TCL and non-treated site. (**B**) Comparison of total area of red blood cells of the control sites without FUS treatment and sites with FUS-treated at different TCLs. The bar plot in (**B**) shows the mean and standard deviation. Each circular point represents the result obtained from each target. (Tukey’s test *P < 0.05; ns: no significant).

The representative T2*-weighted MRI scans acquired at the same position as the T1-weighted MRIs are shown in the bottom column of Fig. 3A. They display no sign of hemorrhage in the FUS-treated site. Figure 4A shows the representative images of H&E-stained whole half brains in the horizontal plane. Histological analysis did not detect evident tissue damage for all TCL groups. Group analysis found no significant difference between the FUS-treated and non-treated groups (Fig. 4B).

## Discussion

This study showed that effective and safe FUS-BBBO was achieved in a porcine model using the proposed real-time feedback control algorithm. Our previous study proposed this algorithm and validated its performance in mice. The current study demonstrated that this algorithm could be scaled up to a large animal model.

Our pig studies found that the performance of the feedback controller was comparable to that of the small animal studies. The averaged stability of the controller as measured by the good pulse rate at 0.5–1 dB ranged from 78.6 to 71.7% in mice and from 84.7 to 64.7% in pigs. Both studies showed a decrease in stability as the TCL increased, indicating decreasing controllability of the cavitation events at higher TCL. Higher TCL requires sonication at higher acoustic pressures. This finding that the stability decreased as TCL increased suggests that microbubble cavitation activity is harder to control at higher acoustic pressures. With regard to the FUS-BBBO outcome, both mouse and pig studies showed that the BBB opening volume monotonically increased when TCL was increased, indicating that the feedback control algorithm can be used to control the FUS-BBBO outcome. As for the safety evaluation, no tissue damage was observed at TCL < 3 dB in mouse studies, and inertial cavitation events and brain tissue damage were observed at TCL ≥ 3 dB in the mouse studies. We did not observe evident tissue damage at TCL ≤ 1 dB in the pig studies. It is noted that 1 dB was selected as the upper limit of the TCL in the pig study. This was because at TCL of 2 dB we observed clear evidence of inertial cavitation and detected obvious tissue damage based on the MRI in one case. We chose 1 dB as the upper limit to avoid causing damage to the pig brain. The finding that damage was observed at TCL ≥ 3 dB in the mouse brains and at 2 dB in the pig brains highlights the importance of selecting the TCL that ensures FUS-BBBO safety in different animal models.

Compared with the feedback controller that is already used in the clinical trials^11–15^, our proposed algorithm has two potential advantages. First, to address the individual difference in cavitation signal among subjects, we defined the TCL relative to the baseline stable cavitation level. In contrast, the algorithm used in the clinical trials defined TCL based on the detection of inertial cavitation, which requires pressure overshooting and thus increases the risk of causing tissue damage. Second, our proposed algorithm provided closed-loop control of the entire FUS-BBBO procedure. In contrast, the algorithms used in published clinical studies were open loop, which maintained at a fixed acoustic pressure after the upper threshold was reached.

This proof-of-concept study demonstrated the feasibility of the proposed feedback control algorithm in controlling FUS-BBBO in a large animal model. Future studies are warranted to further improve its performance. First, the proposed feedback controller only used the stable cavitation level to control FUS-BBBO. Future studies can use the presence of inertial cavitation to set the upper limit for the TCL to maximize BBBO outcome while minimizing the probability of tissue damage. Second, the proposed algorithm can be adapted in the future for controlling FUS-BBBO over a large volume by sonicating multiple targetings in the brain. A survey of baseline cavitation levels over all the target locations can be performed by applying "dummy" FUS sonication at each individual target. Then, the selected TCL for each target location can be defined, and the proposed feedback control algorithm can be used to monitor cavitation activities and control FUS sonication pressure at each target to achieve effective and safe FUS-BBBO over a large volume.

## Conclusions

This study achieved FUS-BBBO using a closed-loop feedback control algorithm in a large animal model. The use of FUS sonication at low acoustic pressure and short duration in the presence of microbubbles to establish the TCL took into consideration individual differences in the detected cavitation signals and avoided overexposure. The proposed feedback control algorithm had high stability and successfully controlled the FUS-BBBO outcomes. Findings from this study demonstrated that this algorithm could be scaled up to a large animal model.

## Data Availability

The analyzed results used to support the findings of this study are included within the article and the code used to acquire the data of this study are available at https://github.com/ChenUltrasoundLabWUSTL/Public-Feedback-control-in-pigs.git. The raw datasets generated during the study are available for research purposes from the corresponding author on reasonable request.
